# Investigation on Dynamic and Static Modulus and Creep of Bio-Based Polyurethane-Modified Asphalt Mixture

**DOI:** 10.3390/polym17030359

**Published:** 2025-01-28

**Authors:** Biao Han, Yongming Xing, Chao Li

**Affiliations:** 1 College of Civil Engineering, Inner Mongolia University of Technology, Hohhot 010051, China; lichao@imut.edu.cn; 2 School of Science, Inner Mongolia University of Technology, Hohhot 010051, China

**Keywords:** asphalt, polyurethane, dynamic modulus, static modulus, creep

## Abstract

The superior mechanical qualities of polyurethane have garnered increasing attention for its application in modifying asphalt mixtures. However, polyurethane needs to use polyols to cure, and polyols need to be produced by petroleum refining. As we all know, petroleum is a non-renewable energy source. In order to reduce oil consumption and conform to the trend of a green economy, lignin and chitin were used instead of polyols as curing agents. In this paper, a biological polyurethane-modified asphalt mixture (BPA-16) was designed and compared with a polyurethane-modified asphalt mixture (PA-16) and a matrix asphalt mixture (MA-16). The viscoelastic characteristics of the three asphalt mixtures were evaluated using dynamic modulus, static modulus, and creep tests. The interplay between dynamic and static modulus and frequency is examined, along with the variations in the correlation between dynamic and static modulus. The creep behavior of the mixture was ultimately examined by a uniaxial static load creep test. The findings indicate that the dynamic modulus of BPA-16 exceeds those of PA-16 and MA-16 by 8.7% and 30.4% at 25 Hz and −20 °C, respectively. At 25 Hz and 50 °C, the phase angle of BPA-16 decreases by 26.3% relative to that of MA-16. Lignin and chitin, when utilized as curing agents in place of polyol, can enhance the mechanical stability of asphalt mixtures at low temperatures and diminish their temperature sensitivity. A bio-based polyurethane-modified asphalt mixture can also maintain better elastic properties in a wider temperature range. At −20–20 °C, the dynamic and static moduli of BPA-16, PA-16 and MA-16 are linear, and they can be converted by formula at different frequencies. The failure stages of BPA-16, PA-16, and MA-16 are not observed during the 3600 s creep duration, with BPA-16 exhibiting the least creep strain, indicating that lignin and chitin enhance the resistance to permanent deformation in PU-modified asphalt mixes.

## 1. Introduction

Entering the twenty-first century after the development of China’s highway into the fast lane, as of 2023, the total mileage of China’s highway is 5,436,800 km, of which 183,600 km is highway. Asphalt pavement provides driving comfort and construction convenience; therefore, asphalt pavement accounts for the majority of China’s highways [1,2]. However, with the rapid increase in highway traffic in China, ordinary asphalt pavement is increasingly difficult to withstand such heavy vehicle loads. Due to polyurethane having more excellent stiffness and fatigue resistance than ordinary asphalt, polyurethane-modified asphalt has attracted the attention of many researchers [3,4].

Polyurethane (PU) has many advantages as a pavement asphalt modifier. Polyurethane-modified asphalt (PA) pavements have been gradually put into use in some countries in Europe and Asia [5,6,7]. Polyurethane is mainly classified into thermosetting polyurethane, thermoplastic polyurethane and waterborne polyurethane (WPU). Polyurethane prepolymers comprise hard and soft segments [8,9]; an elongation of the soft segments enhances the high-temperature performance of asphalt, while an increase in the proportion of hard segments [10] promotes toughness and fatigue resistance in asphalt. Thermoset polyurethane mixtures were found to have better rutting resistance and water stability properties than asphalt mixtures through chemical and physical tests [11]. Moreover, thermoplastic polyurethane (TPU) was employed to enhance the matrix asphalt, hence improving the damage resistance and longevity of asphalt pavement [12]. Even the isocyanate group and hydrogen bonding in PU can stop the rate of aging [13]. Environmentally friendly WPU improves the low-temperature cracking resistance and high-temperature rutting resistance of asphalt [14], and the excellent elongation at break of the flexible long chains in WPU makes asphalt less likely to crack at low temperatures [15].

PU improves the self-healing ability and tensile and compressive strength of matrix asphalt [16,17], and also has a wide range of applications for bridge deck paving and bridge deck expansion joints [18]. The healing temperature of polyurethane asphalt has the greatest effect on the self-healing index, and the degree of aging has the least effect on the self-healing index [19]. Castor oil-based polyurethane-based glass polymers [20], memory polyurethanes [21], and vanillin-based polyurethanes (V-PUs) [22] all enhance the self-healing efficiency of matrix asphalt, with the highest self-healing rate exceeding 90%. Bridge deck paving often uses epoxy resin mixes, and polyurethane mixes have better strength and moisture sensitivity than epoxy resin mixes under high and low temperature conditions [23,24]. Polyurethane can markedly enhance the bond strength, low-temperature resilience, and waterproofing efficacy of the adhesive layer used in bridge deck waterproofing [25,26]. Steel bridge decks have higher requirements for pavement materials, and acrylate composite polyurethane binder (APUB) was synthesized using polyurethane prepolymer (BDO-PU) and acrylate. Dynamic mechanical-thermal analyses, strength experiments, and microscopic tests were used, and it was found that the tensile and compressive properties, water stability, and energy performance of APUB could meet the needs of steel bridge decks relatively well [27]. To address the issue of premature failure of asphalt expansion joints in bridges, three isocyanates and polyols with different ratios of polyurethane were subjected to a series of tests such as initial setting time, fracture strain and aging, and the study proved that PU performed better as expansion joints compared to asphalt, withstanding frequent longitudinal loads and being effective for a longer time under high stress conditions [28].

In addition, there are other aspects of exploration for PA [29]. Polyurethane-modified waste rubber powder asphalt reduces the scattering loss rate of waste rubber powder asphalt and shortens the forming strength time of the mixture [30]. Furthermore, the composite asphalt has better properties than SBR-modified asphalt [31] and can simultaneously solve the problem of asphalt pavement cracking in cold areas in winter and water accumulation in summer [32]. Polyurethane alone mixed with stone can be used as a pavement material and can enhance the splitting strength [33]. Polyurethane is employed to mitigate the adverse impact of waste glass aggregate on asphalt mixtures. Experiments indicate that when the proportions of polyurethane and waste glass aggregate are 10% and 15%, respectively, the strength of the mixture attains its maximum [34]. When polyurethane-modified asphalt is synthesized, the proportion of isocyanate groups is positively correlated with the elastic recovery, viscosity and high-temperature performance of PA and negatively correlated with the low-temperature cracking resistance [35]. To address the deformation and cracking resistance of polyurethane asphalt pavement, 4,4′-disulfonic acid dibisphenol (DSDDP) is used as a chain extender to prepare modified asphalt. The chemical reaction between C-C bond on PU soft section and S-S bond in DSDDP improves the binding force between PU and asphalt macromolecules, thus improving the deformation and cracking resistance [36]. The depolymerization of waste polyethylene terephthalate (PET) produced waste PET polyol, and the modified asphalt prepared with waste PET polyol showed significant improvement in both high-temperature and storage stability [37].

However, polyurethane prepolymers need to use polyols when curing, and polyols are products of petroleum. As we all know, petroleum is a non-renewable energy source. In order to reduce oil consumption in response to the call for a green economy, lignin and chitin are used instead of polyols as curing agents. Marine crustaceans are abundant in chitin [38,39]. Around the world, nearly 10 million tons of shell seafood are treated as garbage every year [40], and even in many backward coastal areas, most of the seafood is landfilled or incinerated by processing plants, causing secondary pollution [41]. Since chitin contains functional groups (-OH, C=O, and -NH_2_), it is also a hot topic in the field of polymers [42,43]. Moreover, chitin can be decomposed into liquid low molecular weight polyols by liquefaction technology, which can recycle seafood waste to save polyols and oil and can effectively promote the solution of global environmental protection issues [44]. Paper mills produce a large amount of discarded lignin every year [45], and in the past, paper mills always sent lignin to power plants for incineration to generate electricity. Since entering the 21st century, science and technology have progressed rapidly, and the value of lignin is far too high for this. The incineration of it not only causes secondary pollution but also has not developed the real value of this natural polymer material. Lignin has low cost and considerable yield, and close to 100 million tons of lignin can be purified from plants by biorefining or pulping technology [46]. There are a lot of hydroxyl groups in lignin, which can be effectively decomposed into polyols after ring-opening to replace the curing agent [47]. Therefore, chitin and lignin have a bright future as renewable natural materials. In addition, the preparation method of polyurethane-modified asphalt and the polyurethane incorporation ratio series also have a significant impact on the performance of modified asphalt. Some studies have proved that the curing reaction is most effective when the polyurethane content is 50% through laboratory experiments and thermochemical and microscopic analysis of the mixture, and the low-temperature performance of the mixture prepared by the internal preparation method is better than that by the external preparation method. The freeze-thaw cycle has little effect on polyurethane-modified asphalt mixture [48].

In summary, most studies on PA focus on the effects of hard segments, soft segments, chain extenders and isocyanates in polyurethane prepolymers on the asphalt ability of PA and do not consider the environmental protection and renewable green economy of polyurethane. There are few studies on the use of chitin and lignin instead of curing agents to synthesize BPA-16; PA-16 and MA-16 were designed for comparison. The viscoelastic characteristics of three asphalt mixtures were evaluated using dynamic modulus, static modulus, and creep tests. The interplay between dynamic and static modulus and frequency is examined, along with the evolving correlation between dynamic and static modulus. Finally, the creep rule of the mixture was studied by the creep test of uniaxial static load.

## 2. Materials and Methods

### 2.1. Materials and Gradations

#### 2.1.1. Raw Materials

**Figure 1:**
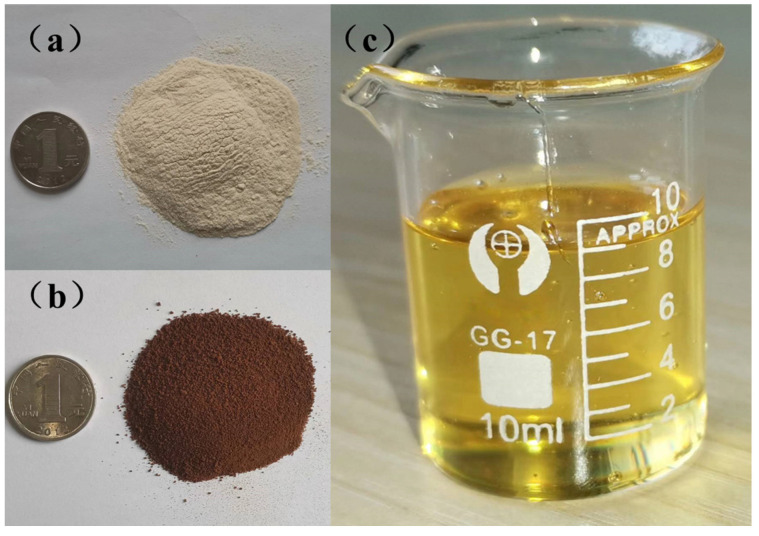
Sample photos: (**a**) Chitin powder; (**b**) Lignin powder; and (**c**) Prepolymer.

Table 1 displays the performance indicators of 90# asphalt, which is sourced from the Panjin Asphalt Factory in China. Chitin was provided by Anhui Kuer Bioengineering Co., LTD. (Hefei, Anhui, China), as shown in Figure 1a; alkali lignin was provided by Beijing Huamaike Biotechnology Co., LTD. (Beijing, China), as shown in Figure 1b; hydrogen peroxide (30%) was purchased from Xiamen Emimani Biotechnology Co., LTD (Xiamen, China). The specific parameters of alkali lignin, chitin and hydrogen peroxide are shown in Table 2. The polyurethane prepolymer is from Shandong Wanhua Chemical Group Co., LTD. (Yantai, Shandong, China), and its performance indexes are presented in Table 3, as shown in Figure 1c. The aggregate of asphalt mixture is basalt, and the filler is limestone mineral powder. The parameters are shown in Table 4 and Table 5. The specific parameters of asphalt and aggregate are listed in JTG E20-2011 [49] and JTG E42-2019 [50], respectively.

#### 2.1.2. Gradations

**Figure 2:**
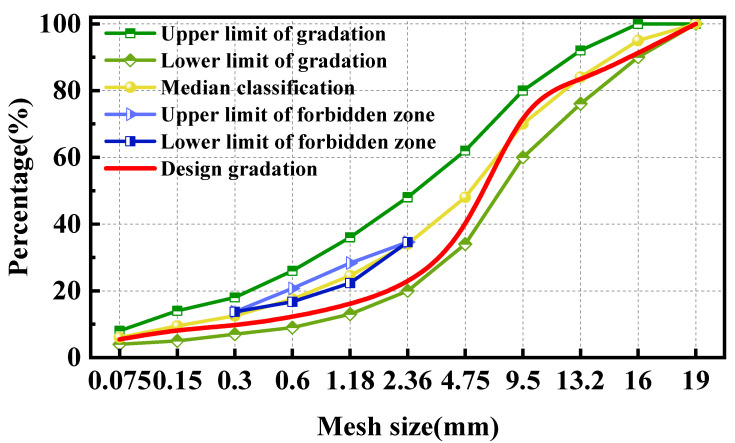
Gradation of mixture.

The three mixtures of BPA-16, PA-16 and MA-16 were graded and designed in accordance with the methods in China’s JTG F40-2017 [51], with a specified maximum particle size of 16 mm. The grading used for the three mixtures is the same, and the grading outcomes are illustrated in Figure 2. The Marshall test results of BPA-16, PA-16 and MA-16 are shown in Table 6, Table 7 and Table 8. In the end, the optimal bitumen content of BPA-16, PA-16 and MA-16 was 5.4%, 5.2% and 4.9%.

### 2.2. Specimen Preparation

In this study, according to the method of Przemysław Bartczak [52], the content of lignin (which needs to be activated with H_2_O_2_ for 30 min before using lignin) and chitin is determined. Through direct stretching experiment and response surface method, and according to the predicted results given by software Design-Expert 13, the maximum TS value can be obtained. The content of lignin was 6.3% (mass percentage of polyurethane prepolymer), and the content of chitin was 3.84%. The content of bio-based polyurethane in BPA was determined by rheological experiment, thermochemical experiment and microscopic test to be 9% (mass percentage of matrix asphalt). This paper provides only a brief overview of the preparation and attributes of bio-based modified asphalt, as discussed in another article by the author.

**Figure 3:**
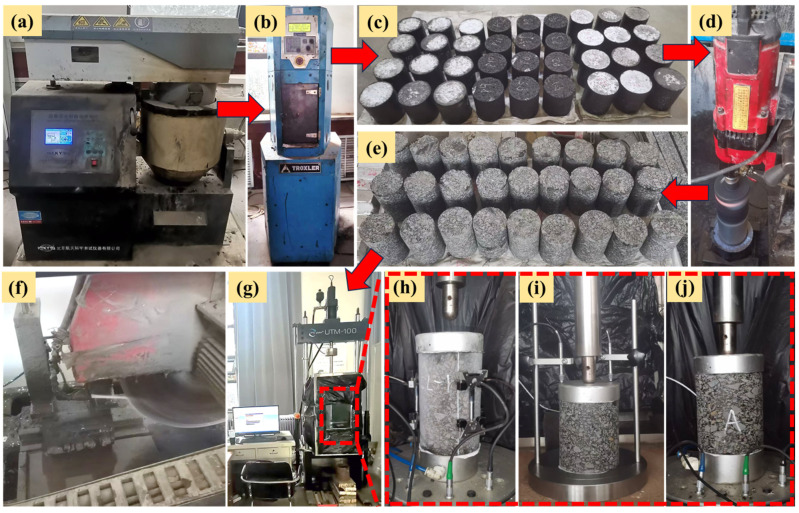
Forming and testing process of asphalt mixture. (**a**) Asphalt mixing pot; (**b**) rotary compactor; (**c**) rotary compactor molding specimen; (**d**) coring machine; (**e**) coring and cut specimens; (**f**) cutting machine; (**g**) UTM-100; (**h**) Dynamic Modulus Test; (**i**) Static Modulus Test; and (**j**) Creep Test.

The preparation process of BPA mixture is carried out at room temperature. First, coarse and fine aggregate, chitin and lignin are placed into the mixing pot for dry mixing for 90 s; subsequently, asphalt and polyurethane prepolymer are poured in for 90 s, and the last step is to add mineral powder and stir for 90 s to mix the mixture evenly, as shown in Figure 3a. Press the mixture into a 170 mm high and 150 mm diameter cylinder using the Gyratory compactor model 5850(Troxler Electronic Laboratories, Durham, North Carolina, USA), as shown in Figure 3b,c. The cylinder specimen was cored (see Figure 3d) and then cut by cutting machine (see Figure 3f) into a cylinder with a height of 150 mm and a diameter of 100 mm (see Figure 3e) for dynamic modulus test. The other part was cut with a size of 150 mm and a diameter of 100 mm and reserved for static modulus and creep tests.

### 2.3. Methods

#### 2.3.1. Dynamic Modulus Test

The loading frequencies were 0.1, 0.5, 1, 5, 10 and 25 Hz, with a total of 6 test frequencies. The region where the corresponding mixture is located is the central region of Inner Mongolia, so six test temperatures are selected: −20, −10, 5, 20, 35 and 50 °C, respectively. The dynamic modulus test was conducted using a universal testing machine (UTM-100, IPC Australia, Sydney, Australia), with the load applied as a half sine wave in accordance with JTG E20-2011 [49]. UTM-100 and the test procedure are shown in Figure 3g,h.

#### 2.3.2. Static Modulus Test

The operating temperatures were selected as −20, −10, 5 and 20 °C according to the central region of Inner Mongolia. A fixture designed for static modulus testing was utilized, with displacement meters fitted on either side to gather deformation data. The static modulus test was carried out by UTM-100. The test process is shown in Figure 3i.

#### 2.3.3. Uniaxial Static Load Creep Test

UTM-100 was used to carry out the creep test under single axial static load. The temperature control of UTM-100 was accurate and met the experimental requirements. The creep deformation of the mixture is very small at low temperature, so the temperatures selected are 20, 35 and 50 °C according to the temperature conditions in central Inner Mongolia. In general, the load of creep test should be 10% of the compressive strength of the mixture. The compressive strengths of BPA-16, PA-16 and MA-16 tested are 19.31, 18.26 and 15.05 MPa, respectively. Therefore, 1.5 MPa was chosen as the creep loading stress. Typically, heavy vehicles carry a load of 1 MPa [53]. Therefore, a stress level of 1 MPa was chosen as a comparison. The loading time of creep test is 3600 s, and the test process is shown in Figure 3j.

## 3. Dynamic Modulus of BPA Mixtures

To assess the modulus variations of BPA-16, PA-16, and MA-16 mixes under dynamic loads, the dynamic moduli of the three mixtures were evaluated at six temperatures and six loading frequencies. The dynamic impacts of matrix asphalt, polyurethane-modified asphalt, and bio-based modified asphalt on asphalt mixes were examined thereafter.

### 3.1. Dynamic Modulus

Dynamic modulus reflects the viscoelasticity and stiffness of asphalt pavement, characterizes the deformation resistance of the mixture under sine-wave stress, and can show the correlation between dynamic modulus and frequency and time [54]. It has been documented that temperature and stress frequency can significantly affect the dynamic modulus of the mixture [55].

#### 3.1.1. Correlation Between Dynamic Modulus and Temperature

**Figure 4:**
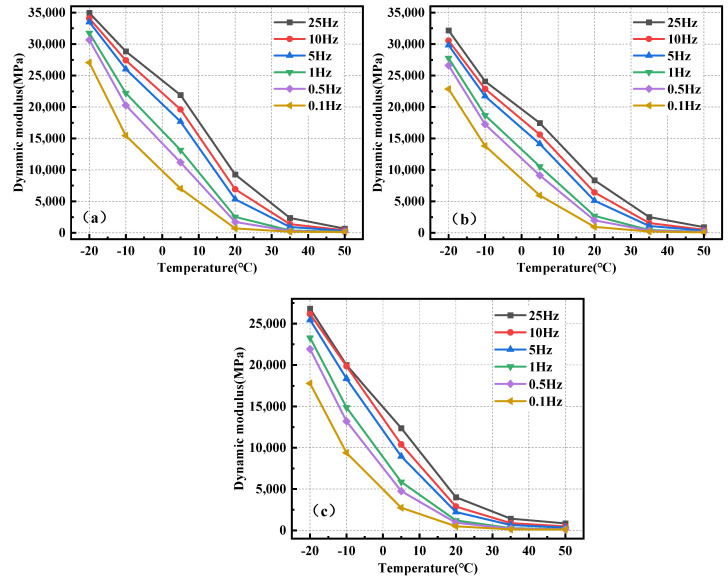
Correlation of dynamic modulus of mixture with temperature. (**a**) BPA-16; (**b**) PA-16; and (**c**) MA-16.

Figure 4 depicts the correlation between the modulus and temperature of BPA-16, PA-16, and MA-16 mixes under dynamic loading conditions.

Figure 4 shows that the dynamic moduli of BPA-16, PA-16 and MA-16 all decrease when the temperature increases at the same frequency, indicating that temperature has a great influence on asphalt mixture, and asphalt exhibits viscoelastic properties at higher temperatures. Under conditions of −20 °C and 25 Hz, the dynamic modulus of BPA-16 attains 35,000 MPa, significantly surpassing its value at 50 °C, indicating that the mixture exhibits elastomeric properties at low temperatures, with a notable enhancement in stiffness. Moreover, at identical temperature and frequency, the sequence of dynamic modulus is BPA-16 > PA-16 > MA-16, signifying that the incorporation of PU has enhanced the high-temperature stability of the matrix asphalt, while the high-temperature stability of the asphalt mixture, achieved by substituting the curing agent with chitin and lignin, has been further augmented.

#### 3.1.2. Correlation Between Dynamic Modulus and Loading Frequency

**Figure 5:**
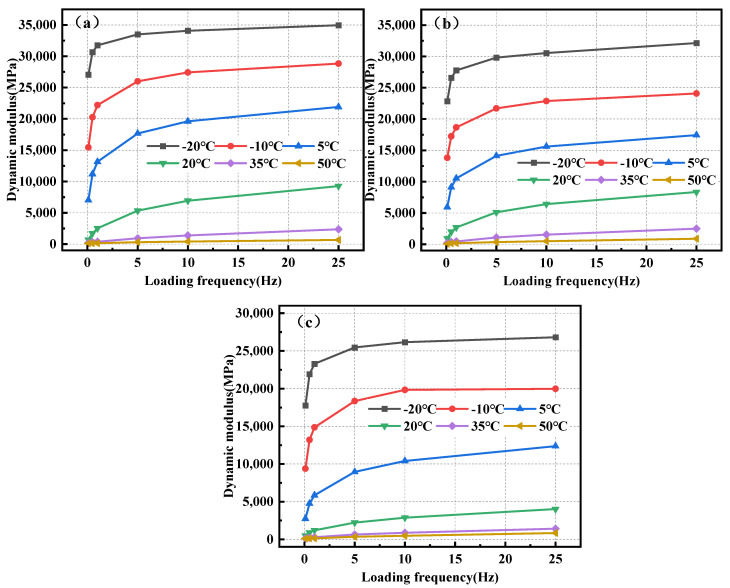
Correlation between the dynamic modulus of the mixture and the loading frequency. (**a**) BPA-16; (**b**) PA-16; and (**c**) MA-16.

Figure 5 illustrates the correlation between loading frequency and the dynamic modulus of BPA-16, PA-16, and MA-16 mixes.

Figure 5 illustrates that the dynamic modulus and frequency of the BPA-16, PA-16, and MA-16 combinations exhibit analogous variations. At temperatures of 20, 35, and 50 °C, both the initial and latter halves of the dynamic modulus exhibit identical behavior with increasing frequency, indicating that the dynamic modulus escalates at a consistent rate. At −20, −10, and 5 °C, the dynamic modulus of the mixture exhibits a pronounced increase at 0.1, 0.5, and 1 Hz initially; however, the rate of increase diminishes at 5, 10, and 25 Hz. This shows that when the load acts on the mixture, the strain response lags behind the stress, which is a characteristic of typical viscoelastic materials, that is, they have rheological properties. Furthermore, the dynamic modulus of BPA-16 exceeds that of PA-16 at temperatures equal to or below 5 °C, indicating that chitin, lignin, and polyurethane collaboratively enhance the low-temperature performance of the mixture.

### 3.2. Phase Angle

The dynamic load applied to asphalt pavement mixture is characterized by the dynamic modulus corresponding to the change of elasticity, while the correlation between the corresponding viscosity is characterized by the phase angle. Elasticity and viscosity collectively represent the anti-deformation capabilities of the mixture under dynamic loading conditions [56].

#### 3.2.1. Correlation Between Phase Angle and Temperature

**Figure 6:**
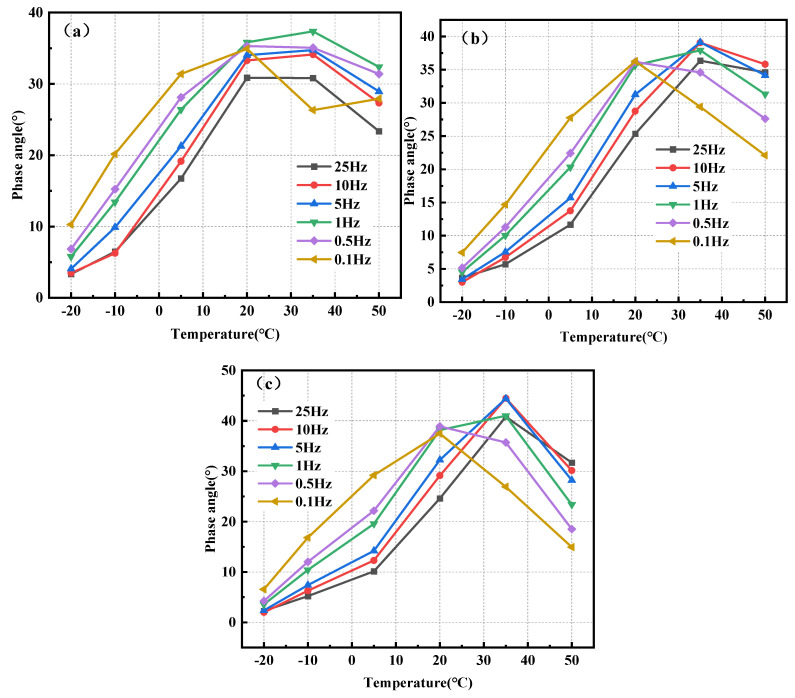
Correlation of phase angle of mixture with temperature. (**a**) BPA-16; (**b**) PA-16; and (**c**) MA-16.

Figure 6 illustrates the correlation between the phase angle and temperature for the three mixtures: BPA-16, PA-16, and MA-16.

The magnitude of the phase angle is inversely correlated with the elasticity of the mixture; a larger phase angle facilitates the production of persistent deformation in the mixture. Figure 6 illustrates that when the temperature rises, the phase angles of the three mixtures initially increase gradually at −20, −10, 5, and 20 °C, indicating elasticity. When above 20 °C, the phase angle under low-frequency load begins to decrease, and viscous characteristics gradually dominate. Furthermore, the phase angle of BPA-16, PA-16, and MA-16 peaked at 35 °C, with BPA-16 exhibiting the lowest phase angle at this temperature, indicating that BPA-16 retains greater flexibility than the PA combination despite elevated temperatures. The incorporation of chitin and lignin enhances the thermal stability of the combination at elevated temperatures.

#### 3.2.2. Correlation Between Phase Angle and Loading Frequency

**Figure 7:**
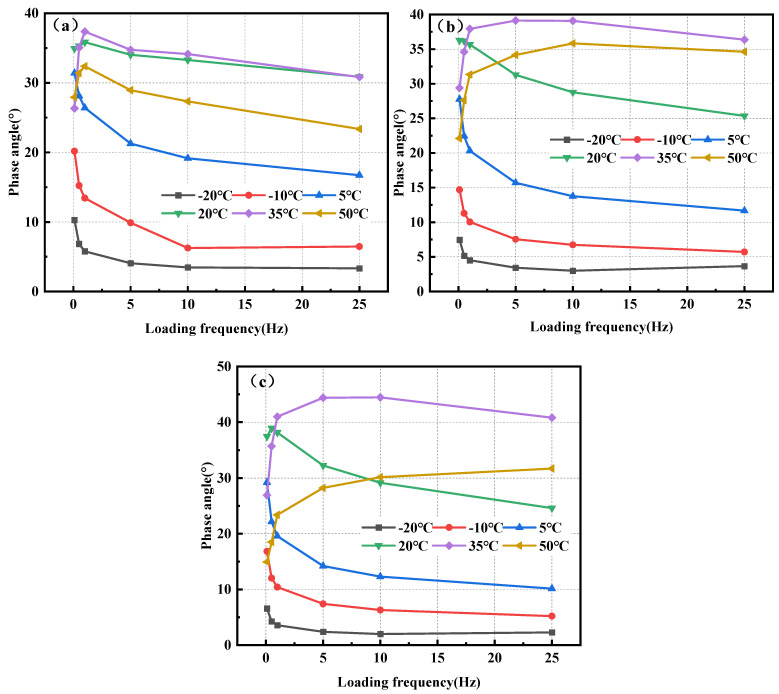
Correlation between the phase angle of a mixture and the loading frequency. (**a**) BPA-16; (**b**) PA-16; and (**c**) MA-16.

The loading frequency inside the mixture can induce deformation in the polymer [57], and there exists a specific correlation between the phase angle of the mixture and the loading frequency. Figure 7 illustrates the correlation between the phase angles and loading frequencies of BPA-16, PA-16, and MA-16.

The three mixtures of BPA-16, PA-16 and MA-16 showed the same rule, and the phase angles of the mixtures gradually decreased at −20, −10 and 5 °C when the loading frequency increased. However, when the mixture is at a temperature of more than 20 °C, the phase angle increases first and then decreases. This shows that when the mixture is less than 20 °C, the mixture under dynamic load is more inclined to elastomer. When temperatures exceed 20 °C, the elasticity of asphalt diminishes while viscosity escalates; hence, an increase in loading frequency does not result in a reduction of the phase angle. Consequently, in comparison to loading frequency, temperature exerts a more significant influence on the viscoelastic characteristics of the combination.

### 3.3. Storage Modulus

The storage modulus is the real component of the complex modulus [58] and signifies the energy retained by a viscoelastic material as a result of elastic deformation during deformation. It denotes the elastic energy density that a material can accumulate under stress, serving as a crucial parameter for characterizing the material’s elastic capacity. The storage modulus is computed as follows:

(1) $$G^{\prime }=G^{*}\times \cos(\phi )$$

where $G^{\prime }$ represents the storage modulus in MPa; $G^{*}$ denotes the dynamic modulus in MPa; ϕ signifies the phase angle.

**Figure 8:**
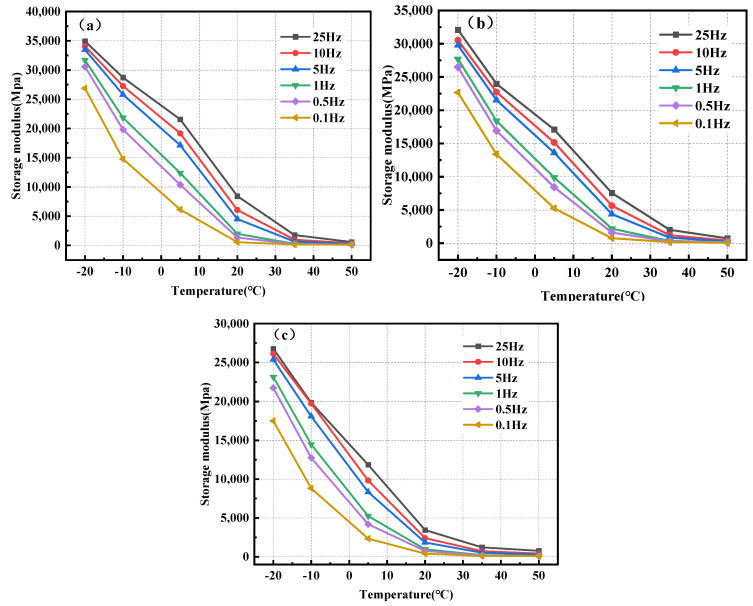
Correlation between the storage modulus of the mixture and temperature. (**a**) BPA-16; (**b**) PA-16; and (**c**) MA-16.

The storage modulus is derived by substituting the dynamic modulus and phase angle obtained from the test into Equation (1). Figure 8 depicts the correlation between the storage modulus and temperature of BPA-16, PA-16, and MA-16. As shown in Figure 8, when the temperature rises, the storage moduli of BPA-16, PA-16 and MA-16 at different frequencies show a trend of narrowing at both ends and widening in the middle. In other words, the storage modulus gap of different frequencies of the mixture is narrowed under low and high temperature conditions, and the storage modulus gap of different frequencies is widened under intermediate temperature conditions. This shows that the properties of the mixture gradually converge to the ideal elastomer at low temperature, and the stiffness of the mixture decreases at high temperature, and its mechanical properties tend to the viscous body. In addition, the storage modulus of bio-based PU-modified asphalt mixture is greater than that of the other two mixtures at the lead-in frequency and temperature, which indicates that chitin and lignin can improve the elastic energy density of PU-modified asphalt mixture.

### 3.4. Loss Modulus

The loss modulus denotes the energy dissipated owing to the irreversible viscous deformation of a material during its deformation, indicating the substance’s viscosity [59]. The loss modulus, also known as the viscous modulus, and the energy storage modulus together form the complex shear modulus.

The calculation formula of the loss modulus is shown in Equation (2):

(2) $$G^{\prime \prime }=G^{*}\times \sin(\phi )$$

where $G^{\prime \prime }$ represents the loss modulus, MPa.

**Figure 9:**
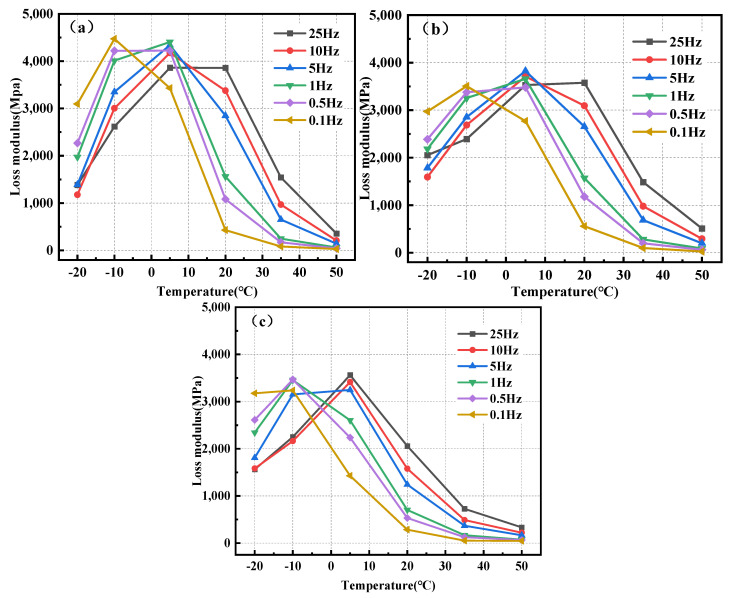
Correlation of loss modulus of mixture with temperature. (**a**) BPA-16; (**b**) PA-16; and (**c**) MA-16.

The loss modulus can be determined by incorporating the dynamic modulus and phase angle received from the test into Equation (2). Figure 9 illustrates the correlation between the loss modulus and temperature for BPA-16, PA-16, and MA-16. Figure 9 illustrates that when the temperature exceeds 5 °C, the loss modulus of the mixture progressively diminishes. Below 5 °C, the loss modulus progressively increases. This validated the principle preceding the phase angle, and the viscosity properties of the combination became evident following the temperature elevation. Moreover, at identical temperature and frequency, the loss modulus of BPA-16 markedly exceeds that of the other two bitumens, suggesting that if equivalent permanent deformation transpires in the three mixtures, the load applied to the BPA-16 mixture will dissipate more energy; thus, BPA-16 exhibits the highest resistance to permanent deformation.

## 4. Dynamic Modulus Master Curve

The dynamic modulus and phase angle of BPA-16, PA-16, and MA-16 combinations exhibit significant variations in response to temperature and loading frequency. Nevertheless, the dynamic modulus throughout a broader spectrum of temperature and loading frequency cannot be acquired due to the limitations of the testing apparatus. Consequently, the primary curve of the mixture’s dynamic modulus can be derived by adjusting the dynamic modulus at various temperatures through the time-temperature equivalence principle [60]. To implement the time-temperature equivalence principle, it is essential to ascertain the shift factor αt and convert the dynamic modulus at the initial frequency to the relevant frequency at the reference temperature, known as the reduced frequency $f_{r}$, as delineated in Equation (3). Simultaneously, the W.L.F equation is employed to determine the shift factor, as seen in Equation (4).

(3) $$f_{r}=f\cdot \alpha_{t}$$

where f is the loading frequency of the test, Hz; $f_{r}$ is the reduction frequency at the corresponding reference temperature, Hz.

(4) $$\mathrm{lg}\left(\alpha_{t}\right)=\frac{-C_{1}\left(t-t_{0}\right)}{C_{2}+\left(t-t_{0}\right)}$$

where $C_{1}$ and $C_{2}$ refer to the material constants determined by fitting; t is the test temperature, °C; $t_{0}$ is the reference temperature, °C.

This article uses the generalized Sigmoidal model [61] to formulate the primary curve of the dynamic modulus, as delineated in Equation (5).

(5) $$\mathrm{lg}\left(\left|G^{*}\right|\right)=\delta +\frac{\alpha -\delta }{{\left(1+\lambda e^{\beta +\gamma \mathrm{lg}f_{r}}\right)}^{\frac{1}{\lambda }}}$$

where |G*| represents the dynamic modulus in MPa; *α* denotes the maximum logarithmic value of the dynamic modulus in MPa; *δ* indicates the minimum pair value of the dynamic modulus in MPa; *β*, *γ*, and *λ* are form factors.

**Figure 10:**
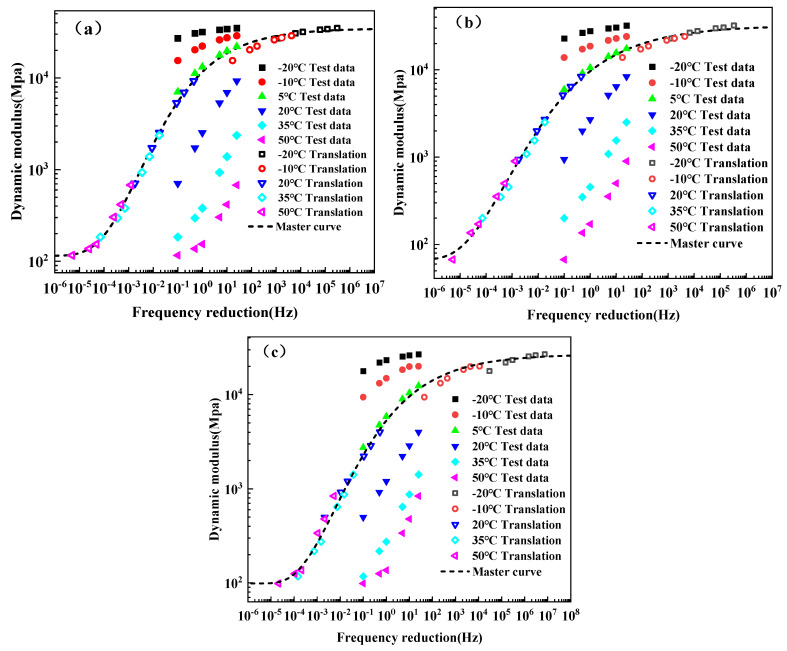
Master curve of dynamic modulus for the mixture. (**a**) BPA-16; (**b**) PA-16; and (**c**) MA-16.

Figure 10 illustrates the primary curves of the dynamic modulus for BPA-16, PA-16 and MA-16 combinations, while Table 9 presents the fitting parameters associated with these curves. The table demonstrates that the decision coefficients for BPA-16, PA-16, and MA-16 exceed 0.99, indicating that the fitted dynamic modulus main curve well represents the variation of dynamic modulus across the extended loading frequency range. The primary curve exhibits a rapid increase in dynamic modulus at low frequencies, followed by a deceleration at high frequencies, which is indicative of the generalized Sigmoidal model. Furthermore, Figure 10 illustrates that as the temperature decreases, the dynamic modulus grows more rapidly with frequency, aligning with the time-temperature equivalence principle.

## 5. Static Modulus of Mixtures

Under the condition of slow loading, that is, when the strain rate is less than 0.1 mm/min, the elastic modulus of the mixture under uniaxial compression is the static modulus [62].

### 5.1. Static Modulus Test Results

When the temperature exceeds 20 °C, the static modulus and dynamic modulus change inconsistently. Therefore, the static modulus is tested after the uniaxial compression strength of the mixture is tested at −20 °C, −10 °C, 5 °C and 20 °C, as shown in Table 10. The static moduli of BPA-16, PA-16 and MA-16 are negatively correlated with temperature, and their static moduli reach their peak value at −20 °C.

### 5.2. Correlation Between Dynamic and Static Modulus

When asphalt pavement is subjected to driving load, the internal force of the mixture is more complicated. Besides vertical load, there are resonance, rebound, damping and horizontal load. Therefore, the viscoelasticity of the mixture under the actual road condition can better reflect the real state of the mixture by using the dynamic modulus.

**Figure 11:**
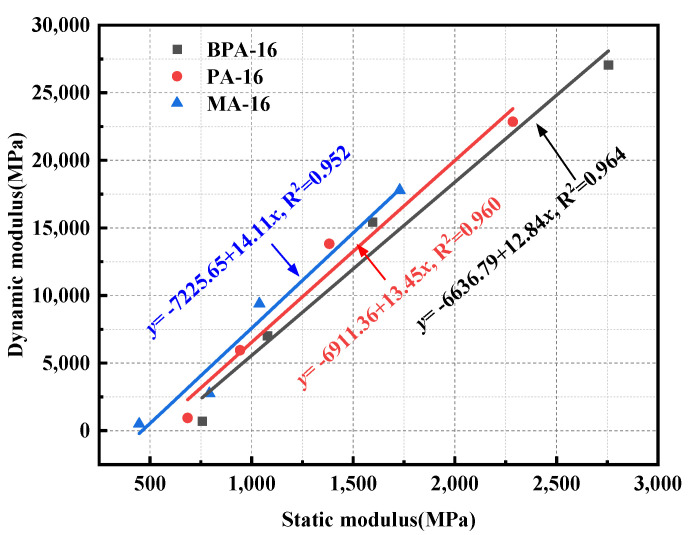
Dynamic modulus and static modulus fit result.

The dynamic modulus has been analyzed in prior tests within this study, yielding the principal curves for the dynamic modulus of BPA-16, PA-16, and MA-16, thus facilitating the demonstration of the correlation between the dynamic modulus and the static modulus. To examine the correlation between dynamic modulus and static modulus, the static modulus at −20 °C, −10 °C, 5 °C, and 20 °C is utilized as the horizontal axis, while the dynamic modulus at 0.01 Hz within the same temperature spectrum is employed as the vertical axis, resulting in the construction of the dynamic and static modulus curve, as illustrated in Figure 11.

The analysis of the dynamic and static moduli of BPA-16, PA-16, and MA-16 demonstrates a linear correlation between the two, with coefficients of determination exceeding 0.95 for all three fitted lines, indicating a high degree of reliability in the fitting results. The results of fitting the dynamic modulus curves for frequencies except 0.01 Hz are presented in Table 11. The table demonstrates that the coefficients of determination for the fitted curves across all frequencies exceed 0.90, signifying a robust linear correlation between the dynamic and static moduli at frequencies ranging from 0.01 to 25 Hz, thereby confirming that the conversion between dynamic and static moduli can be achieved through the fitted parameters.

## 6. Creep Properties of the Mixtures

### 6.1. Creep Curve

**Figure 12:**
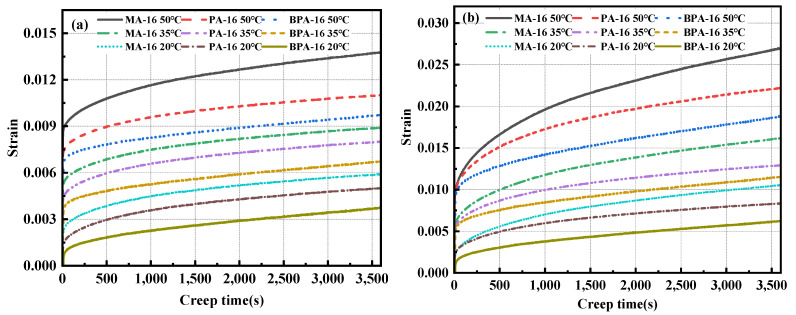
Creep curves under two stresses. (**a**) 1 MPa; (**b**) 1.5 MPa.

The phenomenon that the strain of solid materials increases with the extension of time under the condition that the stress remains unchanged is called creep [63]. Figure 12 illustrates the creep curves of the three mixtures: BPA-16, PA-16, and MA-16. Initially, the strain of the mixture during the early phase of creep accumulates swiftly, followed by rapid compaction of the mixture. Then, the strain growth rate gradually slows down, and the creep curve is in a stable state, which is the second stage. In the end, the mixture did not fail to deform. This shows that the strength of the three mixtures can effectively resist the deformation and failure under the action of persistent load.

As can be seen from Figure 12, when BPA-16, PA-16 and MA-16 have the same gradation and temperature, the order of strain in the creep curve is: BPA-16 > PA-16 > MA-16. This eliminates the effect of mixture grading, fully demonstrating that PU-modified asphalt can improve the resistance of matrix asphalt to continuous load, and bio-based polyurethane-modified asphalt on the basis of PU-modified asphalt further enhances the creep resistance of the mixture.

### 6.2. Creep Compliance Curve

**Figure 13:**
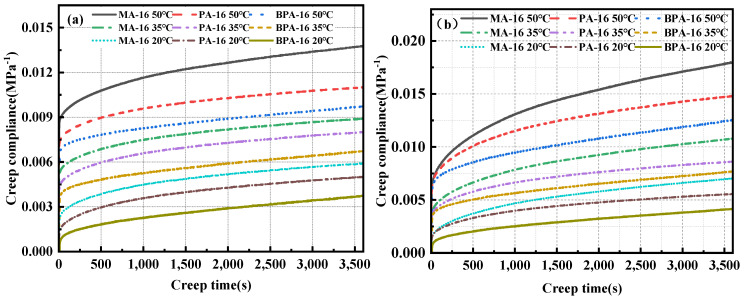
Creep compliance curves under two stresses. (**a**) 1 MPa; (**b**) 1.5 MPa.

Creep compliance denotes a material’s capacity to deform in response to external forces, strongly associated with its stiffness and elasticity [64], and is defined as the ratio of creep strain to stress. Creep compliance characterizes a material’s capacity to resist permanent deformation, with its value being inversely proportional to this resistance. The calculation formula of creep compliance is shown in Equation (6), and the creep compliance curve can be obtained by calculating creep, as shown in Figure 13.

(6) $$J\left(t\right)=\frac{\varepsilon \left(t\right)}{\sigma }$$

where $J\left(t\right)$ denotes creep compliance; $\varepsilon \left(t\right)$ represents the strain; σ signifies the loading stress, in MPa.

Figure 13 illustrates that the creep compliance curve adheres to a comparable principle. Under identical conditions, the creep compliance of BPA-16 is the lowest, whereas that of MA-16 is the highest. BPA-16 exhibits the highest resistance to permanent deformation. Furthermore, when the loading time escalates, the resistance to permanent deformation of the three mixes diminishes gradually. The creep flexibility of BPA-16, PA-16, and MA-16 enhances when the continuous load stress rises to 1.5 MPa, indicating that creep stress significantly affects the material’s resistance to permanent deformation.

## 7. Conclusions

This study presents the analysis of the dynamic modulus, static modulus, and creep tests conducted on BPA-16, PA-16, and MA-16, leading to the following conclusions:

1. At a frequency of 25 Hz and a temperature of −20 °C, the dynamic modulus of BPA-16 increases by 8.7% and 30.4% compared to PA-16 and MA-16, respectively, indicating that the use of lignin and chitin as curing agents, in lieu of polyols, enhances the mechanical stability of asphalt mixtures under low-temperature conditions;
2. Under the condition of 25 Hz and 50 °C, BPA-16 phase angle than MA-16 was reduced by 26.3%, proving that BPA-16 has low temperature sensitivity, explaining that bio-based polyurethane-modified asphalt mixture in a wider temperature range can maintain better elasticity mechanics performance;
3. The dynamic modulus master curves for three mixtures, BPA-16, PA-16, and MA-16, were constructed using the generalized Sigmoidal model based on the idea of time-temperature equivalence. The dynamic modulus may be forecasted across a broad spectrum of temperatures and loading frequencies utilizing the master curves;
4. With BPA-16, PA-16 and MA-16, three maximum static moduli of asphalt mixture under the condition of temperature is 20 °C, measured, and the size of the static modulus sort of BPA-16 > PA-16 > MA-16, lignin and chitosan mass, reduce the temperature sensitivity of asphalt mixture;
5. Between −20°C and 20 °C, it was found that BPA-16, PA-16 and MA-16 three mixture of dynamic and static linear correlation, dynamic and static modulus can be transformed using formulas under different frequencies;
6. As the creep time, temperature and the increase of the creep stress, creep BPA-16, PA-16, and MA-16 three mixture creep strain increase, in the 3600 s the creep time of the destruction of the three kinds of mixture phase does not appear, and the creep strain of BPA-16 minimum, The results showed that lignin and chitin further improved the permanent deformation resistance of polyurethane-modified asphalt mixture;
7. This paper opens up ideas and provides data support for finding bio-based alternatives to polyols for polyurethane curing agents; lignin and chitin are inexpensive, while polyols are derivatives of non-renewable energy sources, i.e., petroleum, which responds to the current trend of green recycling economy and low-carbon emission reduction, and will have great socio-economic value if it can be effectively promoted.

## Figures and Tables

**Table 1:** Performance index of the matrix asphalt.

Performance Index	Unit	Examination Outcomes	Standard
Penetration (25 °C)	0.1 mm	86	80–100
Ductility (15 °C)	cm	>100	≥100
Solubility	v	99.71	≥99.5
Softening point	°C	47.3	≥45
Flash point	°C	281	≥245
Kinetic Viscosity (60 °C)	Pa·s	182	≥160

**Table 2:** Physical parameters of lignin, chitin and H_2_O_2_.

Species	Technical Index	Examination Outcomes
Lignin	Average molecular weight	10,000
	PH	5.5–7.5
	Sulfur content	<3.6%
Chitin	Source	Shrimp shell
	Particle size	<20 μm
H_2_O_2_	Concentration	30%

**Table 3:** Performance index of PU.

Performance Index	Unit	Examination Outcomes	Standard
Isocyanato (–NCO) content	%	16.53	15.6–16.6
Viscosity (25 °C)	MPa∙s	2862	2500–4500
Density (25 °C)	g/cm^3^	1.09	-
Exterior	-	Brown liquid	Liquid

**Table 4:** Specifications of the coarse aggregate.

Performance Index	Unit	Examination Outcomes	Standard
Water absorption	%	0.92	≤2
Bulk specific density	g·cm^3^	2.796	-
Los Angeles abrasion loss	%	20.1	≤28
<0.075 mm particle content	-	0.5	≤1
Apparent gravity	g·cm^3^	2.891	≥2.6

**Table 5:** Specifications of the fine aggregate.

Performance Index	Unit	Examination Outcomes	Standard
Water absorption	%	0.88	-
Bulk specific density	g·cm^3^	2.701	-
Sand equivalent	%	70	≥60
Apparent gravity	g·cm^3^	2.754	≥2.50

**Table 6:** BPA-16 Marshall test results.

BPA Content (%)	Bulk Density (g/cm^3^)	Void Ratio (%)	Saturation (%)	Marshall Stability (kN)	Flow Value (mm)
3.9	2.439	7.1	56.7	18.65	1.6
4.4	2.461	5.4	67.1	21.32	1.8
4.9	2.488	4.3	74.6	24.52	2.0
5.4	2.492	3.6	76.7	26.19	2.1
5.9	2.479	2.5	84.5	25.47	2.2
Standards	-	3–4	75–85	≥6.0	-

**Table 7:** PA-16 Marshall test results.

BPA Content (%)	Bulk Density (g/cm^3^)	Void Ratio (%)	Saturation (%)	Marshall Stability (kN)	Flow Value (mm)
3.7	2.438	7.3	55.6	16.32	1.8
4.2	2.467	6.0	66.1	18.25	1.9
4.7	2.489	4.9	74.1	21.64	2.1
5.2	2.498	3.9	75.6	22.19	2.2
5.7	2.481	3.0	83.4	20.37	2.3
Standards	-	3–4	75–85	≥6.0	-

**Table 8:** MA-16 Marshall test results.

BPA Content (%)	Bulk Density (g/cm^3^)	Void Ratio (%)	Saturation (%)	Marshall Stability (kN)	Flow Value (mm)
4.9	2.475	3.2	76.7	11.91	3.1
Standards	-	3–4	75–85	≥6.0	2–4

**Table 9:** Master curve fitting parameters.

Mixture Type	Model Parameters							R Square
	C_1_	C_2_	α	δ	β	γ	λ	
BPA-16	16.13	123.64	2.06	4.54	−1.05	−0.13	−0.78	R^2^ > 0.99
PA-16	15.74	120.31	1.827	4.51	−1.54	−0.49	−0.14	R^2^ > 0.99
MA-16	13.17	110.25	1.99	4.43	−1.01	−0.49	−0.01	R^2^ > 0.99

**Table 10:** Static modulus test results.

Temperature	BPA-16 (MPa)	PA-16 (MPa)	MA-16 (MPa)
−20 °C	2705	2285	1778
−10 °C	1544	1382	1088
5 °C	1027	943	842
20 °C	706	685	497

**Table 11:** Dynamic and static modulus fitting results.

Type	Loading Frequency	25	10	5	1	0.5	0.1
BPA-16	Intercept	6836.71	3944.90	1653.06	−2890.14	−4425.20	−6636.79
	Slope	11.31	12.08	12.69	13.57	13.63	12.84
	R square	0.901	0.908	0.921	0.952	0.932	0.964
PA-16	Intercept	2328.67	404.14	−1334.08	−4798.91	−5772.62	−6911.36
	Slope	13.73	13.94	14.37	14.90	14.74	13.45
	R square	0.956	0.951	0.96	0.971	0.956	0.960
MA-16	Intercept	−2594.99	−4238.03	−5436.030	−7289.46	−7599.87	−7225.65
	Slope	17.49	18.13	18.24	17.68	16.93	14.11
	R square	0.965	0.957	0.967	0.978	0.963	0.952

## Data Availability

All data generated or analyzed during this study are included in this published article.
